# Developmental exposure to diesel exhaust upregulates transcription factor expression, decreases hippocampal neurogenesis, and alters cortical lamina organization: relevance to neurodevelopmental disorders

**DOI:** 10.1186/s11689-020-09340-3

**Published:** 2020-12-16

**Authors:** Toby B. Cole, Yu-Chi Chang, Khoi Dao, Ray Daza, Robert Hevner, Lucio G. Costa

**Affiliations:** 1 Department of Environmental and Occupational Health Sciences, University of Washington, Seattle, WA USA; 2 Center on Human Development and Disability, University of Washington, Seattle, WA USA; 3 Gradient Corporation, Seattle, WA USA; 4 Department of Pathology, University of California at San Diego, San Diego, CA USA; 5 Center for Integrative Brain Research, Seattle Children’s Research Institute, Seattle, WA USA; 6 Department of Neurological Surgery, University of Washington, Seattle, WA USA; 7 Department of Medicine and Surgery, University of Parma, Parma, Italy

**Keywords:** Air pollution, Diesel exhaust, Developmental neurotoxicity, Adult neurogenesis, Cortical lamina organization, Autism spectrum disorder

## Abstract

**Background:**

Exposure to traffic-related air pollution (TRAP) during development and/or in adulthood has been associated in many human studies with both neurodevelopmental and neurodegenerative diseases, such as autism spectrum disorder (ASD) and Alzheimer’s disease (AD) or Parkinson’s disease (PD).

**Methods:**

In the present study, C57BL/6 J mice were exposed to environmentally relevant levels (250+/−50 μg/m^3^) of diesel exhaust (DE) or filtered air (FA) during development (E0 to PND21). The expression of several transcription factors relevant for CNS development was assessed on PND3. To address possible mechanistic underpinnings of previously observed behavioral effects of DE exposure, adult neurogenesis in the hippocampus and laminar organization of neurons in the somatosensory cortex were analyzed on PND60. Results were analyzed separately for male and female mice.

**Results:**

Developmental DE exposure caused a male-specific upregulation of *Pax6*, *Tbr1*, *Tbr2*, *Sp1*, and *Creb1* on PND3. In contrast, in both males and females, Tbr2^+^ intermediate progenitor cells in the PND60 hippocampal dentate gyrus were decreased, as an indication of reduced adult neurogenesis. In the somatosensory region of the cerebral cortex, laminar distribution of Trb1, calbindin, and parvalbumin (but not of Ctip2 or Cux1) was altered by developmental DE exposure.

**Conclusions:**

These results provide additional evidence to previous findings indicating the ability of developmental DE exposure to cause biochemical/molecular and behavioral alterations that may be involved in neurodevelopmental disorders such as ASD.

## Background

In utero and early childhood exposure to traffic-related air pollution (TRAP) has been associated with neurodevelopmental diseases such as autism spectrum disorder (ASD), attention-deficit/hyperactivity disorder, and obsessive-compulsive disorder ([4, 23, 30, 50, 59, 62, 67]), and with neurodegenerative diseases such as Alzheimer’s disease (AD) and Parkinson’s disease (PD) ([7, 12–14, 24, 25, 43, 63];). We have previously shown that mice exposed developmentally to diesel exhaust (DE) from embryonic day 0 (E0) to postnatal day 3 or 21 (PND3 or PND21) exhibit ASD-like behavioral changes and cortical lamina disorganization [17, 18], as also seen in children with ASD [57]. Other animal studies have also reported behavioral [2, 21, 44, 65] and biochemical [8, 9] effects in rodents following developmental exposure to air pollution. In mice exposed prenatally to DE, differential DNA methylation was reported in promoter regions of genes involving neuronal differentiation and neurogenesis pathways [61].

Air pollution exposure has also been shown to inhibit adult neurogenesis in mice. Our group has shown inhibition of adult neurogenesis in both the sub-ventricular zone (SVZ) and in the hippocampal dentate gyrus (DG) of adult mice exposed acutely to DE [19, 23, 25]. Impaired adult neurogenesis, particularly in the hippocampal sub-granular zone (SGZ), can have important consequences on learning and memory, and has been shown in a number of studies to contribute to the risk of Alzheimer’s disease [1, 32, 42, 46, 66, 70]. Adult neural progenitor cells (NPCs) in the SVZ and SGZ are derived from a slowly dividing subpopulation of embryonic NPCs, whereas fast-dividing embryonic NPCs are responsible for the peak of neurogenesis during CNS development [31, 56]. Environmental toxicants that disrupt the balance between self-renewal and differentiation of NSCs during development could affect the progression of both neurodevelopmental and neurodegenerative disorders.

During CNS development, such processes are controlled in part by the transcription factors paired box 6 (Pax6), T-box brain 2 (Tbr2), and T-box brain 1 (Tbr1), which are expressed sequentially by radial glia, intermediate progenitor cells, and postmitotic neurons, respectively [28]. In adult neurogenesis, the SVZ and hippocampal SGZ contain similar progenitor cells and a similar temporal pattern of expression of *Pax6*, *Tbr2*, and *Tbr1* [11, 39, 52, 53]. Pax6 has been shown to play an essential role in controlling the balance between neural stem cell self-renewal and neurogenesis in both in vivo and in vitro models [33, 45, 54] and regulates the generation of laminae in the cerebral cortex [34]. In Pax77 mice, a transgenic mouse model that overexpresses *Pax6*, expression of both *Tbr2* and *Tbr1* is increased in the fetal brain, and overproduction of Tbr2-positive cells was observed at E12.5, followed by microcephaly at E14.5 [54]. Thus, overexpression of *Pax6* in a transgenic model leads to increased neurogenesis, compromising the self-renewal potential of NPCs early in development. Additionally, Tbr2 has been reported to play an essential role in regulating laminar fate during cortical genesis [48].

In the present study, we investigated the sex-specific effects of developmental DE exposure on expression of *Pax6*, *Tbr2*, and *Tbr1*, and its long-lasting consequences on both cortical lamina organization in the somatosensory cortex and adult neurogenesis in the hippocampal SGZ. The findings have relevance to hippocampal learning and memory, and also provide further evidence of a possible link between air pollution exposure, altered cortical development, and ASD.

## Methods

### Animals and exposure

Nine-week-old male and female C57BL/6 J mice were obtained from the Jackson Laboratory (Bar Harbor, ME) and housed in the University of Washington Northlake Diesel Exposure Facility under specific-pathogen-free conditions on a 12-h light/dark cycle in an Allenton caging system (Allenton, NJ, USA) connected to filtered air supply with unrestricted access to food and water. The current study was performed simultaneously with our previously published studies [17, 18], with the litter used as the statistical unit, and with mice in the current study representing littermates of the mice that underwent behavioral, biochemical, and histological tests in the previous two studies. Following one week of acclimation, each male was time mated with two females overnight, and evidence of a vaginal plug, confirmation of successful mating, was checked the next morning before 8:30 AM. Female mice with identified vaginal plugs were considered to be at embryonic day 0 (E0). Plug-positive females were assigned randomly to Allentown cage racks designated for either diesel exhaust (DE) or filtered air (FA) exposure groups. Pregnant females were housed individually throughout the entire duration of pregnancy, and after birth the dam and litter continued to be housed throughout the pre-weaning period (E0-PND21), until euthanasia at either PND3 or until weaning. Exposure was for 6 h a day, five days per week (Monday through Friday) at a PM_2.5_ concentration of 250+/−50 μg/m^3^, equivalent to a time-weighted hourly average of 44.64-53.51 μg/m^3^. Mice housed in the FA rack were supplied with HEPA-filtered air. In order to keep the exposure schedule consistent on the same developmental days in different cohorts, mating pairs were always set-up on Sundays to allow the first day of exposure to fall on E0.

DE was generated on site from a Yanmar YDG5500 diesel generator fueled with standard highway-grade No. 2 diesel fuel obtained from local fuel distributors and operated under load. Generated DE then passed through a two-step dilution system with dynamic control of fine particulate matter (PM_2.5_), maintaining constant exposure levels at 250+/−50 μg/m^3^. Chemical composition and particle size characterization of the DE have been previously described in detail [29, 68]. During the exposure period, mice in both exposure groups (DE or FA) were housed in the same room under identical conditions, subjected to the same noise level and light cycle. All animal experiments were approved by the University of Washington Institutional Animal Care and Use Committee.

### Pregnancy outcomes, body weights, and postnatal behavioral testing

The current study was part of a larger study involving 13 FA- and 14 DE-exposed litters that were generated over 12 overlapping exposure cohorts. Mice for the current study were littermates of the mice used for behavioral, biochemical, and histological measures in our previous studies [17, 18]. Both DE- and FA-exposed pups were born in similar litter size and sex ratio [17]. There were no significant differences in pup weights, and the appearance of the righting reflex was the same for both DE- and FA-exposed pups [17]. There were no significant differences in maternal behavior between the DE- and FA-exposed dams during the early postnatal period, including time spent on pup-care activities such as grooming, nursing, and nest building [17]. Postnatal behavioral testing of littermates of the mice used in the current study has been reported previously and includes significant effects on social behaviors, communication, and repetitive behaviors [17].

### Tissue collection

On PND3, male and female pups (one per litter) born to DE- and FA-exposed dams were euthanized by rapid decapitation with scissors, and cortex samples were rapidly dissected and snap-frozen in liquid nitrogen, then stored at −80 °C for later quantitative real-time PCR (qRT–PCR) analysis. On PND60, additional mice (one per sex per litter) were euthanized with CO_2_ followed by cervical dislocation, and perfused transcardially with 10 ml of phosphate-buffered saline (PBS) followed by 10 ml of 4% paraformaldehyde at the rate of 2 ml/min. Brains were then carefully removed from the skull, placed into 4% paraformaldehyde at 4 °C overnight for additional fixing then cryoprotected in 30% sucrose at 4 °C until the brains sank. After cryoprotection, brains were hemisected at midline and embedded in Tissue-Tek* CRYO-OCT cutting matrix (Fischer Scientific, Pittsburgh, PA) with midline facing the bottom of a standard-size Cryomold® (25 × 20 × 5 mm, Sakura Finetek, Netherlands) to ensure a consistent sectioning angle. The embedded brains were stored in −80 °C in moisture-trapping resealable zip bags for later immunohistochemistry analysis.

### Quantitative real time-PCR

Levels of mRNA of *Pax6*, *Tbr1*, *Tbr2*, *Sp1*, and *Creb1* were measured in the cerebral cortex of DE- and FA-exposed PND3 mice. In brief, RNA was extracted by homogenizing frozen brain samples in TRIzol reagent (Thermo Fisher Scientific, Rockford, IL) with a tissue homogenizer followed by standard chloroform extraction and ethanol precipitation procedures. RNA was further purified with the GeneJET RNA purification kit (Thermo Fisher Scientific Inc., Rockford, IL) according to the protocol provided by the manufacturer. Quality and concentration of RNA samples were confirmed by NanoDrop (Thermo Fisher Scientific Inc., Rockford, IL) measurements. For qRT-PCR analysis, only samples with 260/280 ratio > 1.8 and 260/230 ratio between 2.0 and 2.2 were used. Reverse transcription was performed using the iScript cDNA Synthesis kit (Biorad; Hercules, CA) with 1 μg of RNA per 20 μl reaction. The iTaq™ Universal SYBR® Green One-Step Kit (Biorad; Hercules, CA) was used for signal detection during qRT-PCR on a Bio-Rad CFX384 Real-Time PCR Detection System (Biorad; Hercules, CA), using the primers indicated in Supplemental Table 1. Expression of target genes was normalized to the housekeeping gene *GAPDH*, and relative expression levels (ddCq) were calculated according to Haimes and Kelley [36]. Expression levels in DE-exposed animals were compared with same-sex animals exposed to FA.

### Immunohistochemical analysis of cortical lamina organization and of hippocampal neurogenesis

OCT-embedded PND60 mouse brains were cut sagittally at 10-12 μm starting 2000 μm away from the midline, and the somatosensory cortex region was sampled 200 μm apart for five serial sets. The sections were direct-mounted on glass slides and air dried, then stored at −80 °C. Immunohistochemistry was performed as previously described [28] with the following primary antibodies at the indicated dilutions: rabbit anti-Tbr1 (1:2000; obtained from Dr. Robert Hevner), rat anti-Tbr2 (1:200; Ebioscience, 14-4875-82), rabbit anti-CUX1 (1:200; Santa Cruz, sc-13024), rat anti-Ctip2 (1:1,000; Abcam, 18465), mouse anti-Calb (1:3,000; Sigma, c9848), and rabbit anti-Parvalbumin (PV) (1:1,000; Swant, PV27). Secondary antibodies were Alexa Fluor 488-conjugated goat anti-rabbit (1:600, Thermo Fisher Scientific), 568-conjugated goat anti-mouse (1:600, Thermo Fisher Scientific), and 568-conjugated goat anti-mouse (1:600, Thermo Fisher Scientific). The chromosome counterstain DAPI (4′,6-diamidino-2-phenylindole) (Sigma, St. Louis, MO) was used to label DNA after incubation with primary and secondary antibodies, following the manufacturer’s protocol. Brains from five animals in each experimental group were processed and analyzed, with 3-5 sections collected per brain. Digital immunofluorescence images were obtained on a Zeiss Axio Imager Z1. In each image, cortical depth (i.e., distance between the ventricle and pia mater), was divided into 10 evenly spaced bins, with bin 1 nearest the pia mater. Fluorescent-labeled cells were counted in each bin and the area of each bin was measured using Adobe Photoshop, with the researcher blinded to experimental groups. Cell density in each bin was calculated by dividing cell count by bin area.

### Statistical analysis

Statistical analyses for cortical lamina quantifications were performed as previously described [18]. The two-tailed *t* test was used to assess differences in cell density between FA and DE brains of the same sex in each bin. The *F* test was also used to assess equality of variances between FA and DE brains of the same sex in each bin. In the hippocampus, Tbr2+ cells in the DG were counted in 3-5 tissue sections from each of the five mice sampled per experiment group. Averaged Tbr2+ cell counts for each animal were normalized to same-sex FA control. The unpaired *T* test with Welch’s correction was used to compare differences in Tbr2+ cell counts in the hippocampus between FA- and DE-exposed brains of mice of the same sex.

## Results

### Developmental DE exposure is associated with increased expression of the transcription factors *Pax6*, *Tbr2*, *Tbr1*, *Sp1*, and *Creb1* on PND3

**Fig. 1:**
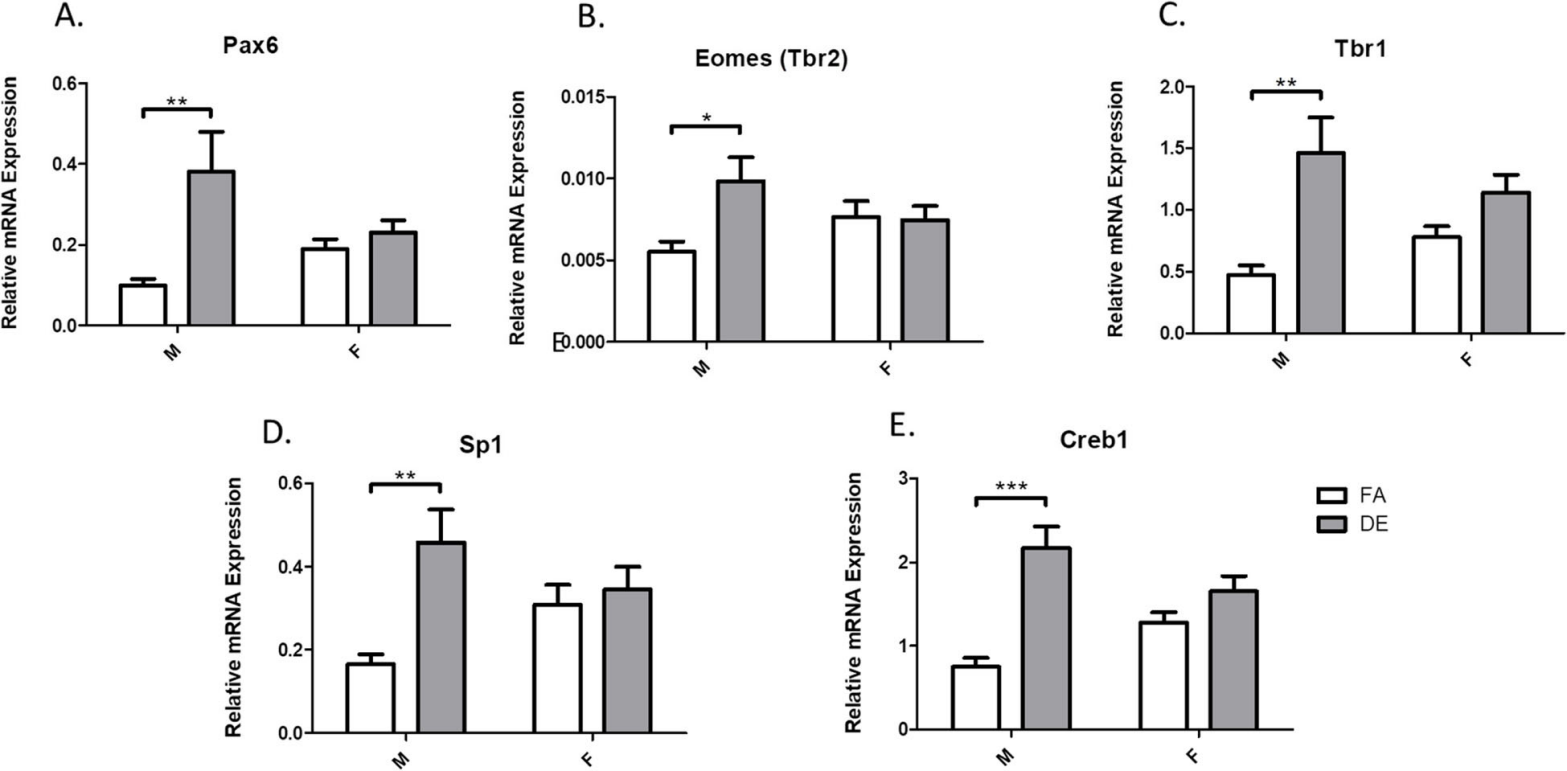
mRNA levels of neurogenic transcription factors at PND3. mRNA levels of transcription factors *Pax6* (**a**), *Tbr2* (**b**), *Tbr1* (**c**), *Sp1* (**d**), and *Creb1* (**e**) were measured in cortex samples from PND3 pups exposed to either DE or FA from E0 to PND3. mRNA levels were normalized to the house-keeping gene *GAPDH*. Results represent the mean (± SE) of 5 mice from different litters for each experimental group. For all five transcription factors measured, mRNA levels were significantly increased in DE exposed males when compared to FA males (*p < 0.05; **p < 0.01; ***p < 0.001; two-way ANOVA with Bonferroni post-test)

Levels of mRNA of transcription factors known to modulate neurogenesis and neuronal differentiation were measured by qRT-PCR in whole brains from PND3 male and female mice exposed to DE or FA from E0 to PND3. The relative expression of each of the target genes *Pax6*, *Tbr2*, *Tbr1*, *Sp1*, and *Creb1* was normalized to the housekeeping gene *GAPDH*. As shown in Fig. 1, expression of all transcription factors increased significantly in brains of DE-exposed male mice compared to FA controls. In female mice, there was a slight trend toward increased expression, particularly for *Tbr1* and *Creb1*, but it was not statistically significant (Fig. 1). There were no differences in *GAPDH* expression between DE- and FA-exposed animals (not shown).

### Developmental DE exposure is associated with decreased adult neurogenesis

**Fig. 2:**
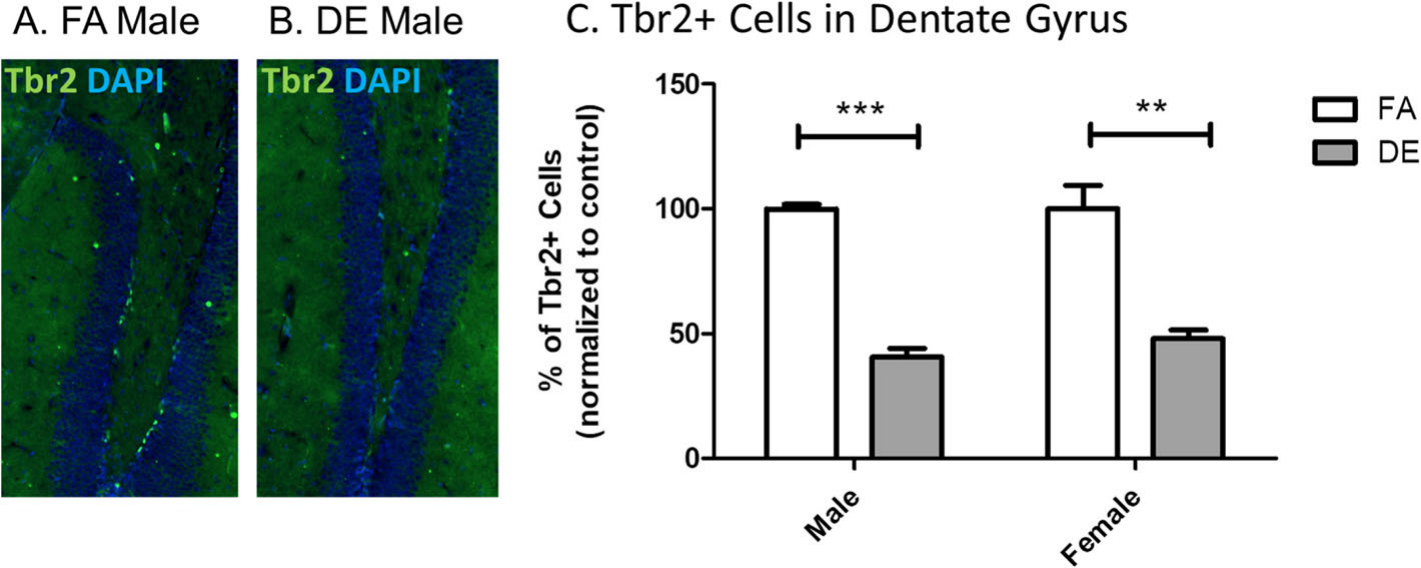
Adult neurogenesis in the hippocampal dentate gyrus. Adult neurogenesis in the hippocampal dentate gyrus, as measured by Tbr2 immunohistochemistry. Tbr2 is expressed in intermediate progenitor cells [28, 39], and in the adult brain is a marker of newly-divided neurons in the subventricular zone (SVZ) and hippocampal subgranular zone (SGZ) [11, 39, 52, 53]. Representative images from a FA-exposed male (**a**) and DE-exposed male (**b**) are shown, with Tbr2+ cells (in green), counterstained with the nuclear stain DAPI (blue). DE exposure was associated with a significant decrease in the number of Tbr2+ cells in the SGZ in both males and females (**c**). Results represent the mean (± SE) of 5 mice from different litters for each experimental group (**p < 0.01; ***p < 0.001; unpaired *t* test with Welch’s correction)

The presence of T-box brain 2 (Tbr2) has been used as a marker for neurogenesis during CNS development and in the adult brain, as it is expressed by intermediate neuronal progenitors [28, 39]. In the adult brain, neurogenesis occurs in two discrete areas, the sub-ventricular zone (SVZ), and the hippocampal sub-granular zone (SGZ) of the dentate gyrus (DG) [1, 66]. At PND60, mice exposed developmentally to DE exhibited a significant decrease in the number of *Tbr2*+ cells in the hippocampal SGZ region, with the decrease seen in both males and females (Fig. 2). Thus, exposure of mice to DE during development was associated with a decrease in hippocampal adult neurogenesis later in life at PND60.

### Disorganization of cortical laminae in PND60 mice exposed developmentally to DE

**Fig. 3:**
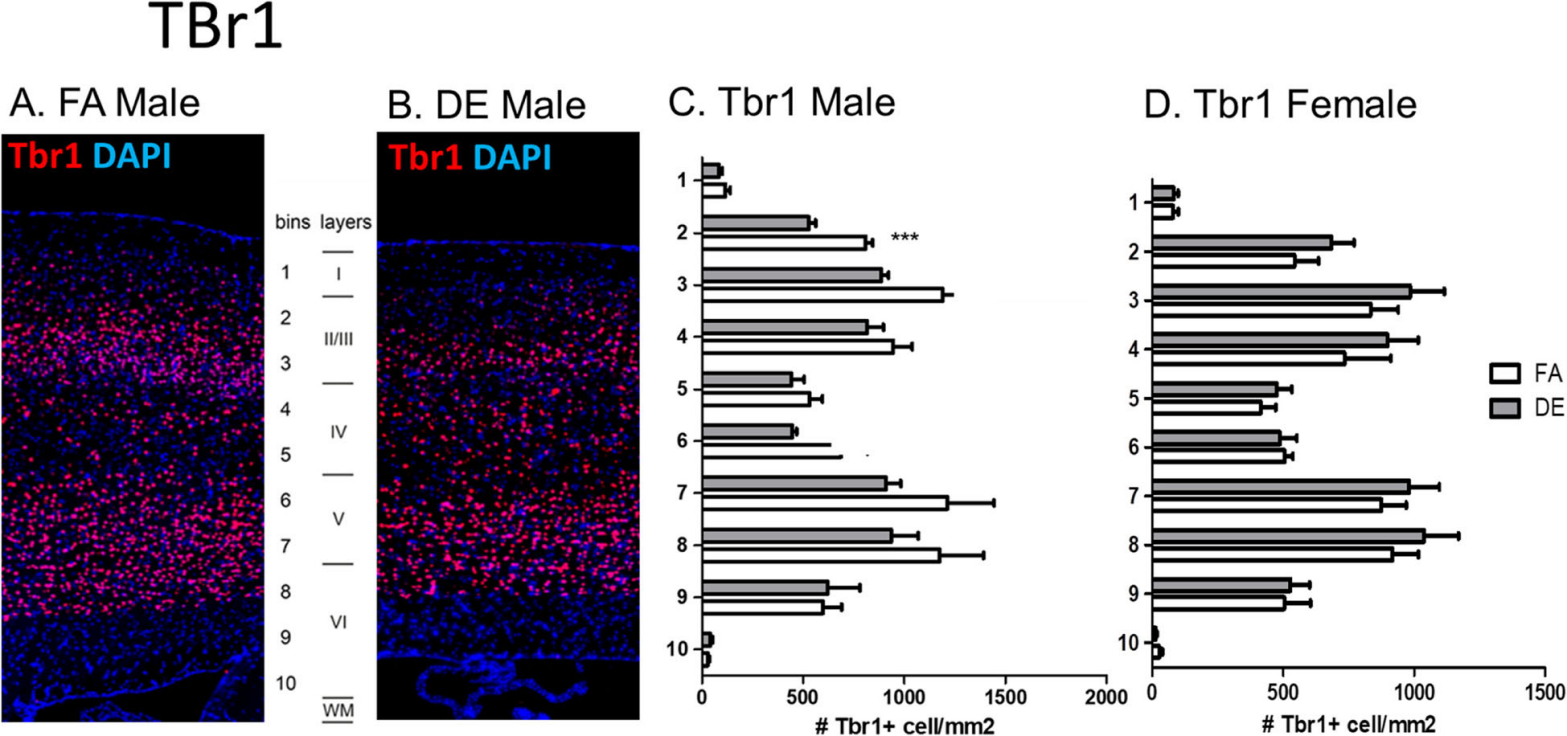
Tbr1 in PND60 cortex. Tbr1 immunohistochemistry in cortex of DE- and FA-exposed mice. Tbr1 is normally localized to neurons in layers II/II and V, as demonstrated in the Allen Brain Atlas [60]. (**a**, **b**) Representative images of Tbr1+ cells (red), counterstained with the nuclear stain DAPI (blue), from FA- and DE-exposed males. DE-exposed males showed a statistically significant decrease in Tbr1-positive cell density in bin 2 (cortical layer II/III) compared to FA-exposed males (**c**). No differences in Tbr1-positive cell distribution were observed with DE exposure in female mice (**d**). Results represent the mean (± SE) of 5 mice from different litters for each experimental group; 3-5 sections/mouse were examined (***p < 0.001; unpaired *t* test with Welch’s correction)

**Fig. 4:**
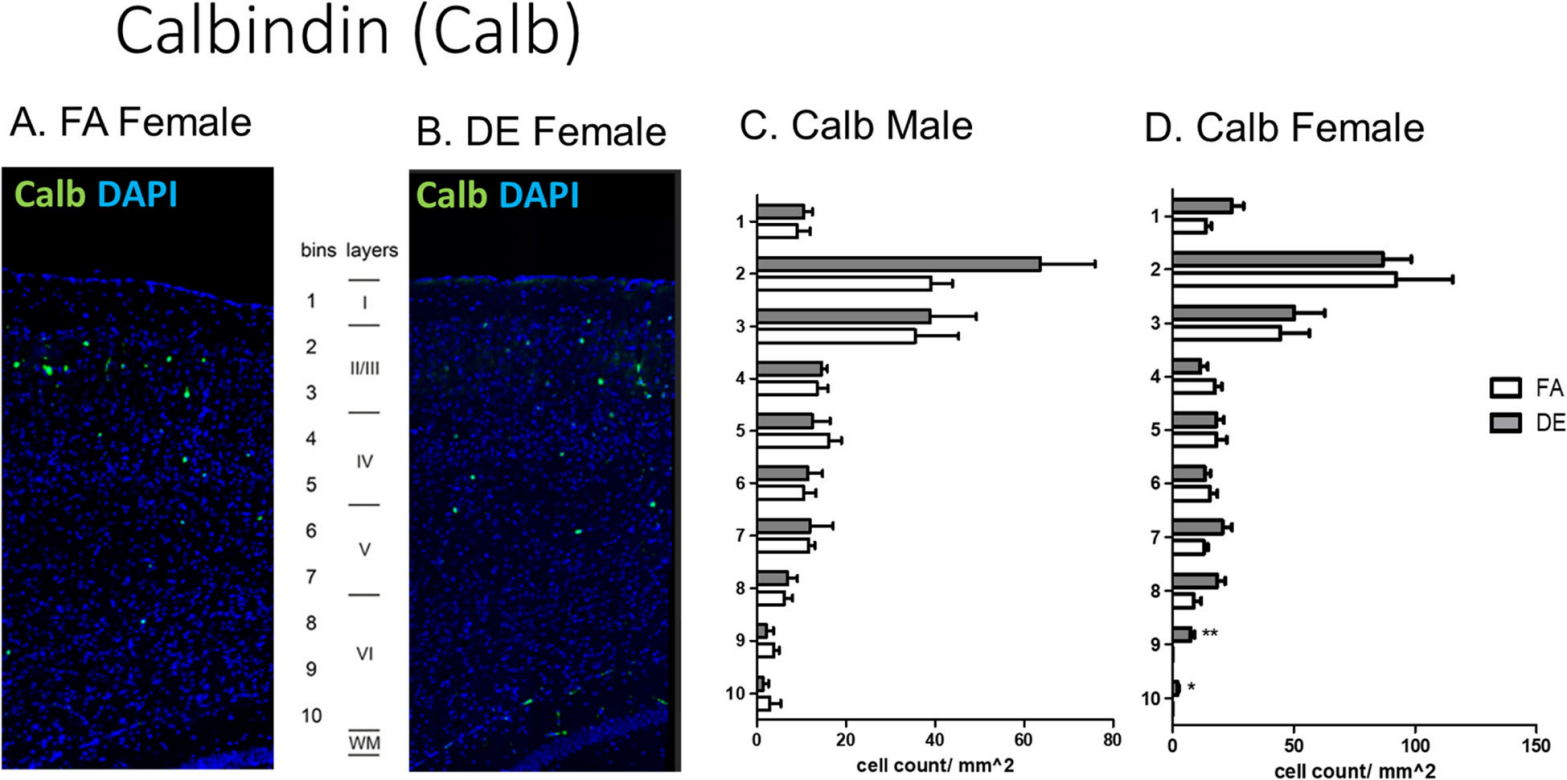
Calbindin in PND60 cortex. Calbindin (Calb) immunohistochemistry in cortex of DE- and FA-exposed mice. Calb is a calcium-binding protein; Calb-expressing interneurons have been shown to localize in cortical layer II/III [5]. (**a**, **b**) Representative images of Calb-positive cells (green), counterstained with the nuclear stain DAPI (blue), from FA- and DE-exposed females. In DE-exposed females, a statistically significant increase in Calb-positive cell density was found in bins 9-10 (cortical layer VI) when compared to FA-exposed females (**c**, **d**). No significant differences in Calb-positive cell distribution were found between FA- and DE-exposed male mice (**c**, **d**). Results represent the mean (± SE) of 5 mice from different litters for each experimental group; 3-5 sections/mouse were examined (*p < 0.05, **p < 0.01; unpaired *T* test with Welch’s correction)

**Fig. 5:**
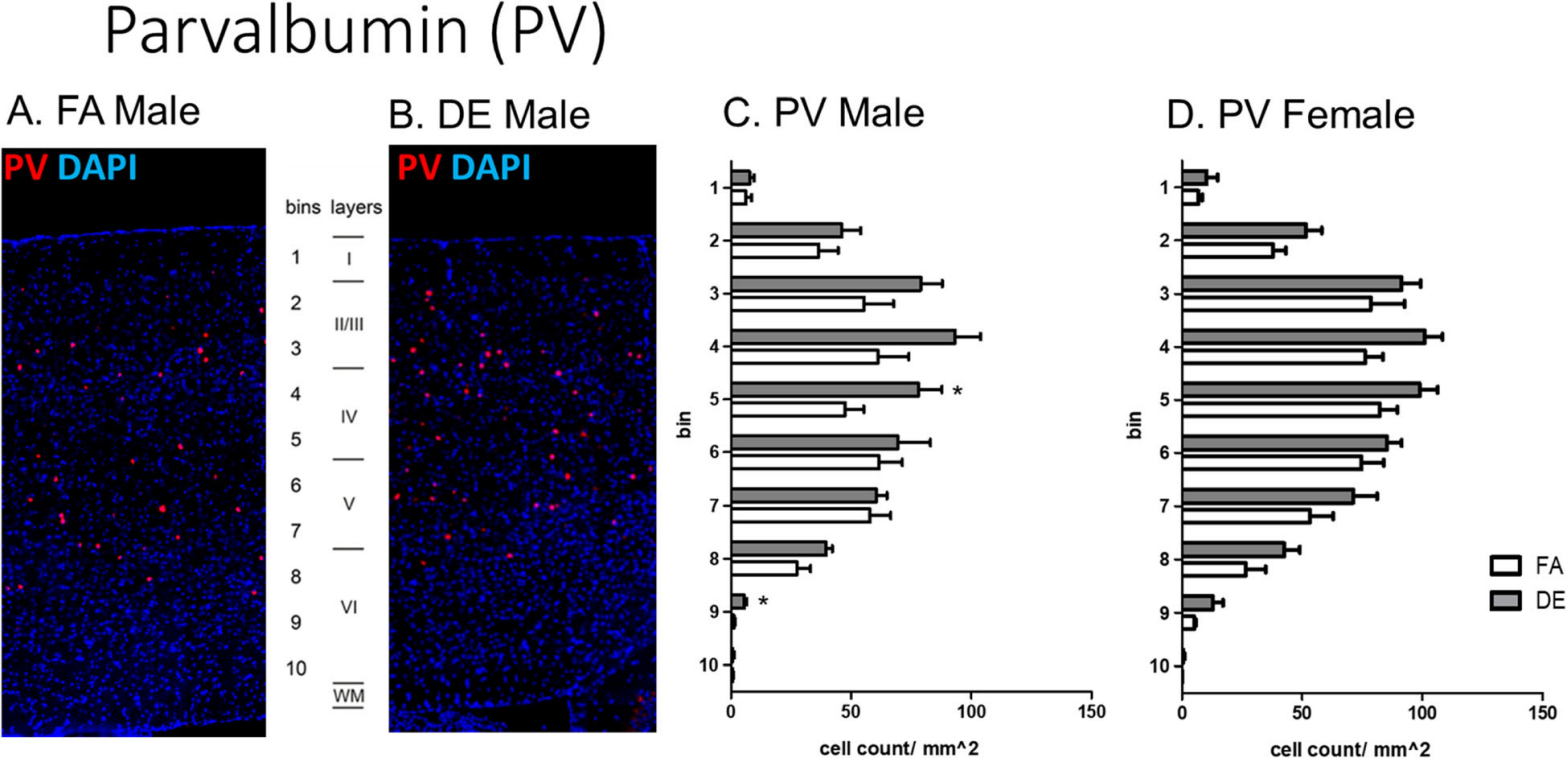
Parvalbumin in PND60 cortex. Parvalbumin (PV) immunohistochemistry in cortex of DE- and FA-exposed mice. PV is a calcium-binding albumin protein expressed by interneurons localized to cortical layer II/III [5, 35]. (**a**, **b**) Representative images of PV-positive cells (red), counterstained with the nuclear stain DAPI (blue), from FA- and DE-exposed males. In DE-exposed males a statistically significant increase in PV-positive cell density was found in bins 5 and 9 (cortical layers IV and VI) when compared to FA-exposed males (**c**, **d**). No significant differences in PV-positive cell distribution were found between FA- and DE-exposed female mice (**c**, **d**). Results represent the mean (± SE) of 5 mice from different litters for each experimental group; 3-5 sections/mouse were examined (*p < 0.05; unpaired *T* test with Welch’s correction)

Disruptions of cerebral cortex development have been demonstrated in mice genetically modified to overexpress *Pax6* [40], as well as in mice exposed prenatally to PM_2.5_ [69] or DE [17]. To assess potential effects on lamina organization due to developmental DE exposure, an immunohistochemical analysis was carried out at PND60 in the somatosensory cortex, using the lamina-specific markers T-box brain 1 (Tbr1), COUP-TF-interacting protein 2 (Ctip2), and CUT-like homeobox 1 (Cux1), as well as Calbindin (Calb) and Parvalbumin (Pv). Statistically significant differences were found in the cortical distribution of Tbr1-, Calb-, and Pv-positive cells. Tbr1 is a transcription factor known for its role in glutamatergic neuron differentiation [28, 49]. Developmental DE exposure affected the localization pattern of Tbr1-positive cells at PND60, with a less distinct bi-laminar pattern than that seen in control mice and a statistically significant decrease in Tbr1-positive cells in cortical layer II/III in the DE-exposed males, but not females (Fig. 3). In contrast, FA-exposed control mice of both sexes and DE-exposed female mice exhibited a distinct bi-laminar localization pattern, with Tbr1-positive cells localizing in layers II/III and V (Fig. 3c, d). Calbindin (Calb) is a calcium-binding protein, and Calb+ interneurons have been shown to localize primarily but not exclusively in cortical layer II/III [5]. In DE-exposed female mice a statistically significant increase in Calb+ cell density was found in cortical layer VI when compared to FA-exposed females, whereas no significant difference in Calb+ cell distribution was found between FA- and DE-exposed male mice (Fig. 4c, d). Parvalbumin (Pv) is a calcium-binding albumin protein expressed by interneurons localizing primarily in layer II/III [5, 35]. In DE-exposed male mice, a statistically significant increase in Pv + cell density was found in cortical layers IV and VI when compared to FA-exposed males, whereas no significant differences in Pv + cell distribution were found between FA- and DE-exposed female mice (Fig. 5c, d). No significant differences were found in the cortical distribution of Ctip2- and Cux1-positive cells following DE exposure (Supplemental Figs. 1 and 2).

## Discussion

The expression of five different transcription factors (*Pax6*, *Tbr2*, *Tbr1*, *Sp1*, and *Creb1*) known to modulate neurogenic pathways [27, 28, 34, 54, 58] were found to be increased in DE-exposed males on PND3 (Fig. 1). PND3 was selected for the analyses because cortical neurogenesis is complete and markers of different neuron types are strongly expressed, enabling analysis of cortical neuron types quantitatively by PCR. Temporal expression of *Pax6*, *Tbr2*, and *Tbr1* has been shown to control the process of neurogenesis and neurodifferentiation, as these transcription factors are expressed sequentially by radial glia, intermediate progenitor cells, and postmitotic neurons, respectively, during neurogenic events in both the fetal and adult brain [11, 28, 39, 52, 53]. In addition, Pax6 has been shown to play an essential role in controlling the balance between neural stem cell self-renewal and neurogenesis in both in vivo and in vitro models [33, 54], and in transgenic mice that overexpress *Pax6*, the expression of both *Tbr2* and *Tbr1* has been shown to be positively regulated [54]. Our findings of increased *Pax6*, *Tbr2*, and *Tbr1* mRNA levels in the cortex of PND3 male mice exposed developmentally to DE (Fig. 1) suggest that DE exposure caused a delay in the differentiation of cortical progenitors and neurons in male mice, since transcription-factor levels normally decline postnatally, but remained elevated after DE exposure. These findings would be consistent with the reported upregulation of *Tbr2* and *Tbr1* expression by Pax6 [54], leading to promotion of neurogenesis at the expense of neural stem cell self-renewal. In the DE-exposed males, expression of *Pax6*, *Tbr2*, and *Tbr1* were all increased, despite their different developmental roles. One possibility to explain this observation is that there could be an overall increased and prolonged abundance of developmental cell types (both progenitor cells and new neurons) in the DE-exposed male mice. If the population of radial glial cells (Pax6+ cells) is increased, they may generate more intermediate progenitors (Tbr2+) and neurons (Tbr1+). The predicted consequence would be that overall neurogenesis, and therefore brain size, is increased. Interestingly, this fits with clinical observations of increased brain volume in a significant proportion of autism cases, marked by brain overgrowth during the late gestational and early postnatal developmental periods [37].

Immunohistochemistry analysis in the hippocampal DG of PND60 mice exposed developmentally to DE (E0-PND21) showed a significant decrease in Tbr2-positive cells in the SGZ of both male and female mice (Fig. 2). Since Tbr2 is only expressed by newly divided intermediate neural progenitor cells [28, 39], this finding suggests that developmental DE exposure decreases adult neurogenesis. Given the importance of the hippocampus for learning and memory, this effect of DE exposure on adult neurogenesis in the SGZ could account for neurodevelopmental problems and even deleterious effects in adults. Other studies have shown decreased adult neurogenesis due to acute and subacute exposure to traffic-related air pollution in adult mice [19, 20], but this long-lasting effect of developmental exposure is novel. In Pax77 mice, a line of transgenic mice that overexpress *Pax6*, an increased number of Tbr-positive cells was observed in the neocortex at E12.5, followed by microcephaly at E14.5 with no increase in apoptosis [54]. Our findings suggest that over-promotion of neurogenesis early in CNS development depletes the self-renewal potential of neural progenitor cells, which leads to decreased neurogenesis during the later stages of corticogenesis, as well as to decreased adult neurogenesis in the SGZ. Alternatively, DE exposure may impair the postnatal migration and integration of neural stem and progenitor cells in the hippocampal SGZ. Consistent with the latter possibility, developmental DE exposure has been shown to affect the expression and distribution of reelin [18], which plays important roles in migration and differentiation of neurons in the cortex and hippocampus [10]. Furthermore, mRNA levels of the transcription factor *Creb1* were also increased in brains of PND3 male mice exposed developmentally to DE (Fig. 1e). As Creb1 is reported to play a role in neuronal survival during adult neurogenesis [38, 71], further assessment of whether *Creb1* upregulation due to DE exposure persists in adult mice would be of interest.

We also found increased mRNA levels of the transcription factor *Sp1* in the brains of PND3 male mice exposed developmentally to DE (Fig. 1d). Elevated expression of *Sp1* has been observed in the brains of autism patients [64], along with altered expression of a number of ASD candidate genes that have Sp1 binding sites, including *reelin* (*RELN*). Increases in *Sp1* expression and downregulation of *RELN* expression were observed in both ASD patients [64] and in the brains of mice exposed developmentally to DE using the same experimental paradigm as the current study [18]. Further, in the gene encoding, the MET receptor tyrosine kinase, a functional promoter variant has been identified that was associated with ASD in two separate studies and that alters Sp1 binding [15, 16]. The diverse downstream pathways mediated by the Sp1-regulated genes, along with the environmental and intracellular signal-related regulation of Sp1, could explain the complex phenotypes associated with ASD. Increased *Sp1* expression is also of interest to neurodegenerative disease, as Sp1 has been found to upregulate the β-amyloid precursor protein (APP) as a result of environmental influences during brain development (e.g., exposure to the neurotoxic metal lead), leading to amyloidogenesis and cognitive decline at a later age [3, 6].

In the somatosensory cortex, we found a significantly decreased number of Tbr1-positive cells in cortical layers II/III from PND60 male mice exposed developmentally to DE (Fig. 3c), similarly to what was found in the Pax77 mouse model, where cortical layers are generated with an “inside-out” pattern, with neurons in deeper layers born during early stages of corticogenesis [41, 51, 55]. This sex-specific finding from the Tbr1 immunohistochemistry analysis is also in agreement with the developmental effects of DE exposure on *Pax6*, *Tbr2*, and *Tbr1* expression levels, which were also male specific (Fig. 1). The male specificity of these effects is of particular interest given the higher incidence of ASD in males and is consistent with our previous studies showing that males were often more sensitive to the behavioral and biochemical effects of developmental DE exposure [17, 18]. It is intriguing that the inhibition of hippocampal adult neurogenesis following developmental DE exposure occurred in both males and females, whereas the cortical effects and effects on ASD-related behaviors were more male-specific. The underlying factors affecting the different sensitivities of males and females are unknown. Adult mice exposed to DE have also shown increased sensitivity of males to DE-associated oxidative stress and neuroinflammation [22].

We have previously reported downregulation of *reelin* (*RELN)* expression in brains of mice at PND3 and PND60 following developmental DE exposure using the same experimental paradigm as the current study [18]. Since reelin has been known to play a critical role in guiding the process of neuronal migration [26, 47], we carried out in the current study an immunohistochemical analysis with four additional cortical lamina markers (Calb, Pv, Ctip2, and Cux1) to further evaluate the extent of potential cortical organizational effects due to developmental DE exposure. We found subtle changes in the distribution of Calb+ and Pv + cells in deeper layers of the cortex (Figs. 4 and 5), while no significant changes in cell distribution were found with the markers Ctip2 and Cux1 (Supplemental Figs. 1 and 2). Although the level of cortical disorganization found in DE-exposed mice is mild compared to that observed in the reelin knockout mouse [10], our results parallel reports of small patches of disorganization found in prefrontal cortex of adolescent ASD patients [57]. The consequences of such subtle effects on cortical layering is not known, but even minor alterations in cortical structure may be of importance, particularly when they occur in a region such as the somatosensory cortex that can be altered in ASD. Our previous studies have demonstrated ASD-related behavioral consequences of developmental DE exposure, including effects on communication, social behaviors, and repetitive behaviors [17]. This would suggest that the subtle effects of DE exposure on cortical layering in the current study have direct phenotypic relevance.

## Conclusions

Our findings show that developmental DE exposure is associated with upregulation of the Pax6, Tbr2, and Tbr1 neurogenic pathway, likely, disrupting the temporal balance between neurogenesis and progenitor cell self-renewal and leading to decreased neurogenesis during late cortical development. Related to this finding, we found that developmental DE exposure from E0-PND21 was associated with decreased adult neurogenesis in the hippocampus at PND60, detected by a decrease in Tbr2-positive cells in the SGZ. This important finding suggests that developmental DE exposure could have long-lasting consequences on learning and memory that extend into adulthood. In addition to this effect on hippocampal adult neurogenesis, we found perturbations in cortical layering of the somatosensory cortex at PND60 that were associated with developmental DE exposure, specifically with respect to neurons expressing Tbr1, Calb, and Pv. These findings are of relevance to neurodevelopmental disorders, and particularly ASD, as our previous findings showed ASD-related behavioral alterations arising after developmental DE exposure. The effects on cortical development are likely due to perturbations of reelin, an important mediator of cortical development that is affected by developmental DE exposure [18]. Taken together, these findings suggest that DE exposure affects postnatal migration and integration of neural stem and progenitor cells in both the SGZ and cortex. Much more still remains unknown about the risk from developmental air pollution in respect to both neurodegenerative diseases and neurodevelopmental disorders. Given the pervasive nature of traffic-related air pollution and the size of populations exposed globally, future studies are warranted to carefully evaluate both the potential hazard and the exact mode of toxicity of TRAP.

## Supplementary Information

**Additional file 1: Supplemental Table 1.** Sequences of primers for qRT-PCR. **Supplemental Figure 2.** Ctip2 in PND60 Cortex. **Supplemental Figure 3.** CUX1 in PND60 Cortex.

## Data Availability

The original data are available at the University of Washington.

## Abbreviations

AD: Alzheimer’s disease
APP: Amyloid precursor protein
ASD: Autism spectrum disorder
Calb: Calbindin
CNS: Central nervous system
Creb1: cAMP-responsive element binding protein 1
Ctip2: Coup-TF interacting protein 2
Cux1: Cut-like homeobox 1
DE: Diesel exhaust
DG: Dentate gyrus
E0: Embryonic day 0
FA: Filtered air
GAPDH: Glyceraldehyde-3-phosphate dehydrogenase
NPCs: Neural progenitor cells
Pax6: Paired box 6
PD: Parkinson’s disease
PV: Parvalbumin
PND: Postnatal day
SGZ: Subgranular zone of the hippocampal dentate gyrus
Sp1: Specificity protein 1
SVZ: Subventricular zone
Tbr1: T-box brain 1
Tbr2: T-box brain 2
TRAP: Traffic-related air pollution
