# Supplementary material for: Developmental exposure to diesel exhaust upregulates transcription factor expression, decreases hippocampal neurogenesis, and alters cortical lamina organization: relevance to neurodevelopmental disorders
Source: J Neurodev Disord. 2020 Dec 16;12:41. doi: 10.1186/s11689-020-09340-3 (PMC7745370; doi:10.1186/s11689-020-09340-3)
Supplement: Supplementary file 1 — Additional file 1: Supplemental Table 1. Sequences of primers for qRT-PCR. Supplemental Figure 2. Ctip2 in PND60 Cortex. Supplemental Figure 3. CUX1 in PND60 Cortex. [file 11689_2020_9340_MOESM1_ESM.zip › Cole J Neurodev Disord Suppl Material TC102519.docx]

|  | Forward Primer Sequence (5' -> 3') | Reverse primer Sequence (5' -> 3') |
| --- | --- | --- |
| PAX6 | ACTTTAACCAAGGGCGGTGAG | TTCACTCCGCTGTGACTGTTC |
| Tbr1 | CCGGAGACTCAGTTCATCGC | GCCCGTGTAGATCGTGTCAT |
| Tbr2 | GTGACGGCCTACCAAAACAC | CCACCTCTTCGCTGAATCGT |
| Sp1 | AGGCCTCCAGACCATTAACC | TCCATGATCACCTGGGGTGT |
| Creb1 | ACTCAGCCGGGTACTACCAT | GAGGCAGCTTGAACAACAACT |
| GAPDH | TGACCTCAACTACATGGTCTACA | CTTCCCATTCTCGGCCTTG |

**Supplemental Table 1.** Sequences of primers for qRT-PCR

Sequences of primer sets used in the present study are shown; both forward and reverse primers are listed from 5’ to 3’.

**Supplemental Figure 1. Ctip2 in PND60 Cortex.**

COUP-TF-interacting protein 2 (Ctip2) is expressed in cortical layer V as described in the Allen Brain Atlas (Sunkin et al., 2013). (A, B) Representative image of Ctip2+ cells (green), counterstained with the nuclear stain DAPI (blue), from FA- and DE- exposed males. Developmental DE exposure was not associated with any statistically significant differences in Ctip2+ cell distribution in either males or females (C, D). Results represent the mean (± SE) of 5 mice from different litters for each experimental group; 3-5 sections/ mouse were examined; Statistical significance was determined by unpaired T-test with Welch’s correction.

**Supplemental Figure 2. CUX1 in PND60 Cortex.**

CUT-like homeobox 1 (CUX1) is expressed in cortical layers II/III and IV as described in the Allen Brain Atlas (Sunkin et al., 2013). (A, B) Representative image of CUX1+ cells (red), counterstained with the nuclear stain DAPI (blue), from FA- and DE- exposed males. Developmental DE exposure was not associated with any significant differences in CUX1+ cell distribution in either males or females (C, D). Results represent the mean (± SE) of 5 mice from different litters for each experimental group; 3-5 sections/ mouse were examined. Statistical significance was determined by unpaired T-test with Welch’s correction.
