# Supplementary figures and images for: Developmental exposure to diesel exhaust upregulates transcription factor expression, decreases hippocampal neurogenesis, and alters cortical lamina organization: relevance to neurodevelopmental disorders
Source: J Neurodev Disord. 2020 Dec 16;12:41. doi: 10.1186/s11689-020-09340-3 (PMC7745370; doi:10.1186/s11689-020-09340-3)

## Slide 1
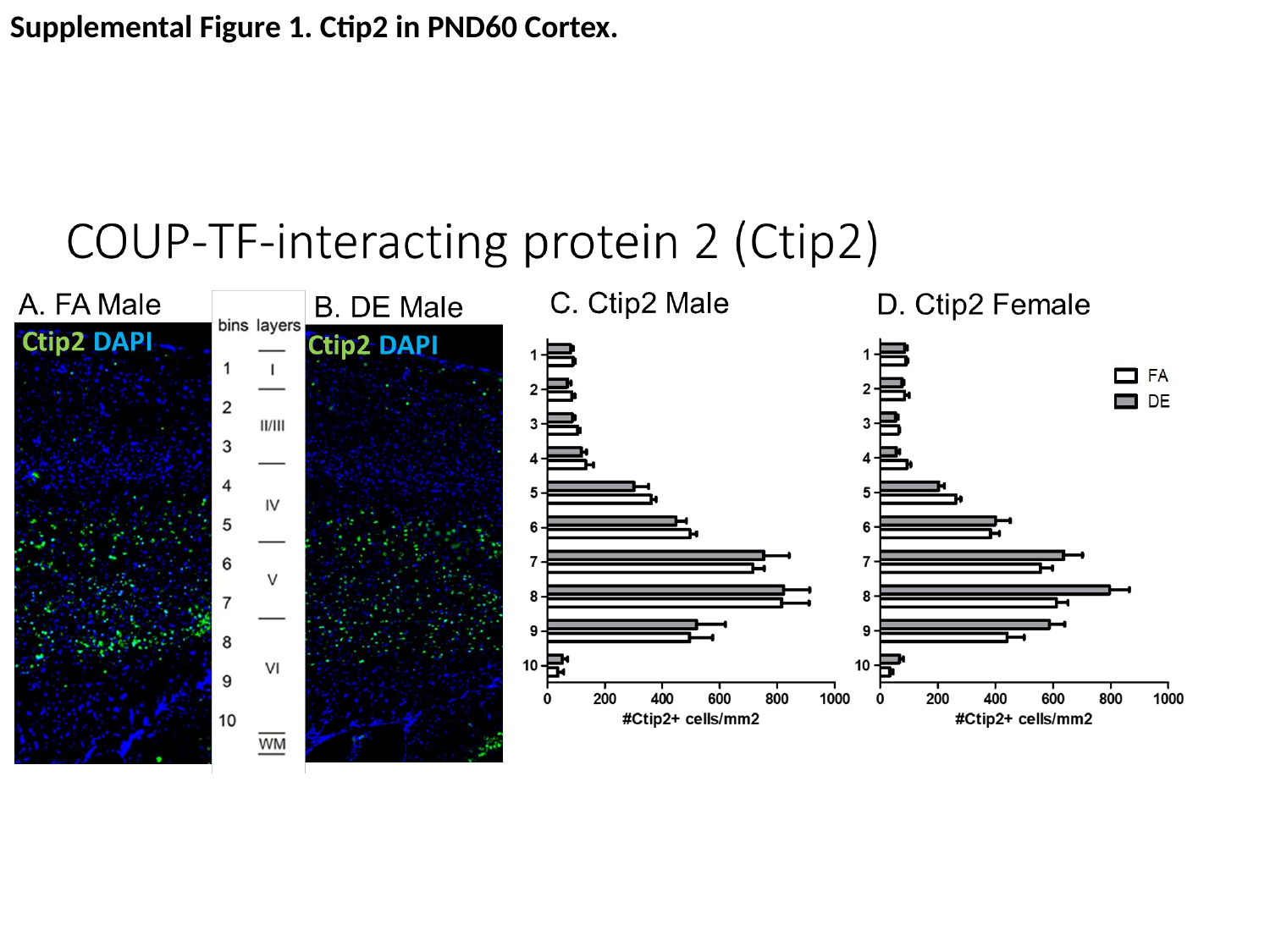

Supplemental Figure 1. Ctip2 in PND60 Cortex.

Supplement: Supplementary file 1 — Additional file 1: Supplemental Table 1. Sequences of primers for qRT-PCR. Supplemental Figure 2. Ctip2 in PND60 Cortex. Supplemental Figure 3. CUX1 in PND60 Cortex. [file 11689_2020_9340_MOESM1_ESM.zip › Suppl Fig 1.pptx]

## Slide 1
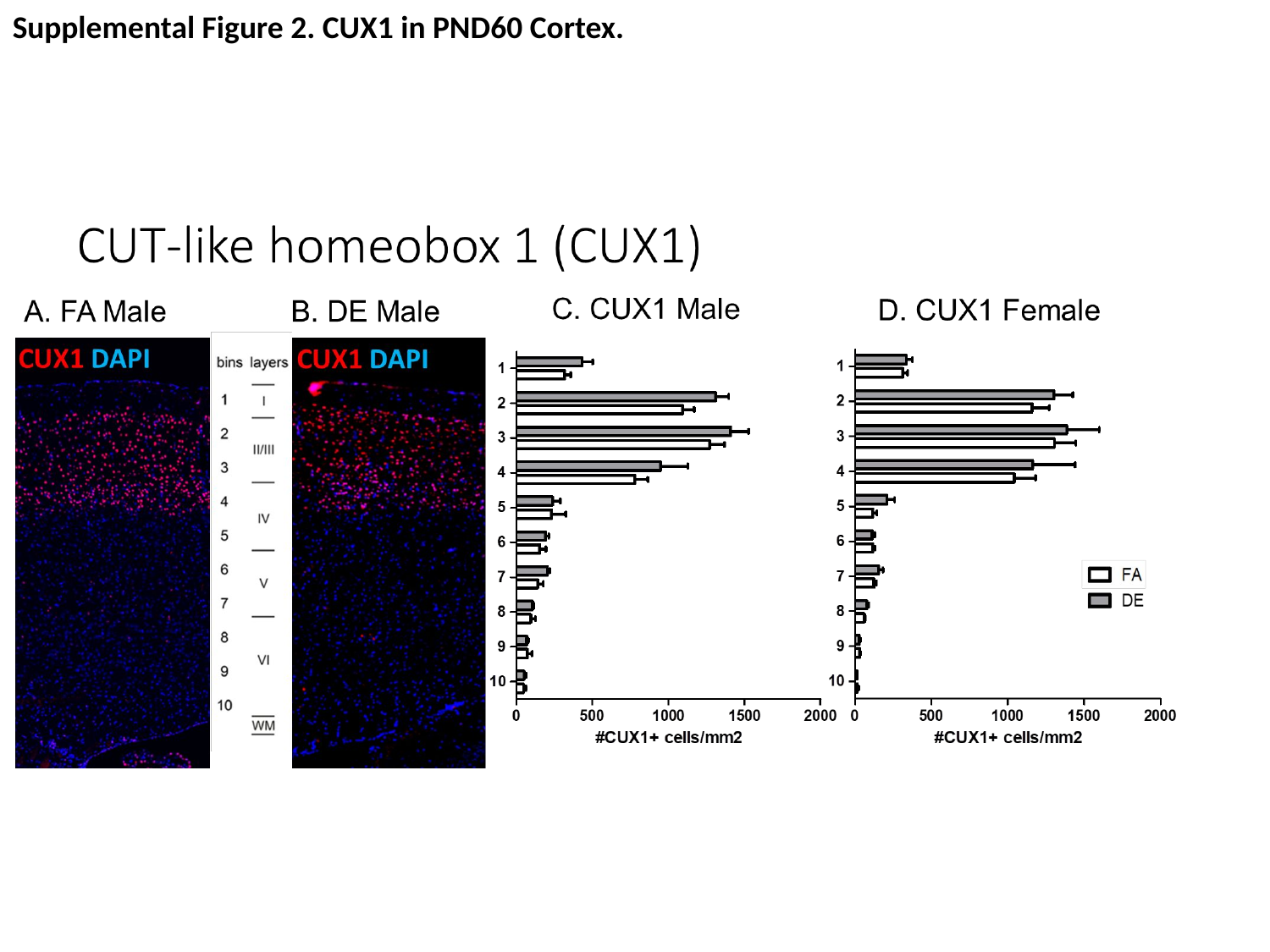

Supplemental Figure 2. CUX1 in PND60 Cortex.

Supplement: Supplementary file 1 — Additional file 1: Supplemental Table 1. Sequences of primers for qRT-PCR. Supplemental Figure 2. Ctip2 in PND60 Cortex. Supplemental Figure 3. CUX1 in PND60 Cortex. [file 11689_2020_9340_MOESM1_ESM.zip › Suppl Fig 2.pptx]
